# The epidemiological characteristics and molecular phylogeny of the dengue virus in Guangdong, China, 2015

**DOI:** 10.1038/s41598-018-28349-2

**Published:** 2018-07-02

**Authors:** Jiufeng Sun, Huan Zhang, Qiqi Tan, Huiqiong Zhou, Dawei Guan, Xin Zhang, Jinhua Duan, Songwu Cai, Zhiqiang Peng, Jianfeng He, Changwen Ke, Jinyan Lin, Tao Liu, Wenjun Ma, De Wu

**Affiliations:** 1 Guangdong Provincial Institute of Public Health, Guangdong Provincial Center for Disease Control and Prevention, Guangzhou, 511430 China; 2 Key Laboratory for Repository and Application of Pathogenic Microbiology, Research Center for Pathogens Detection Technology of Emerging Infectious Diseases, Guangdong Provincial Center for Disease Control and Prevention, Guangzhou, 511430 China; 3 WHO Collaborating Centre for Surveillance, Research and Training of Emerging Infectious Diseases, Guangzhou, 511430 China

## Abstract

In 2015, an unexpected multiple outbreak of dengue occurred in Guangdong, China. In total, 1,699 cases were reported, of which 1,627 cases were verified to have DENV infections by nucleic acid or NS1 protein, including 44 DENV-1, 1126 DENV-2, 18 DENV-3 and 6 DENV-4, and the other cases were confirmed by NS1 ELISA. Phylogenetic analyses of DENV-1 isolates identified two genotypes (I and V). The predominant DENV-2 outbreak isolates were the Cosmopolitan genotypes, which likely originated from Malaysia. The DENV-3 isolates were assigned into genotype I and genotype III. All 6 DENV-4 isolates from imported cases were likely originally from Cambodia, Thailand and the Philippines. The entomological surveillance showed a moderate risk for the BI index in Chaozhou and Foshan and a low risk in Guangzhou. The imported cases were mostly detected in Guangzhou and Foshan. Surprisingly, the most serious outbreak occurred in Chaozhou, but not in Guangzhou or Foshan. A combined analyses demonstrated the multiple geographical origins of this outbreak, and highlight the detection of suspected cases after the alerting of imported cases, early implementation of control policies and reinforce the vector surveillance strategies were the key points in the chain of prevention and control of dengue epidemics.

## Introduction

Dengue virus (DENV) is an enveloped flavivirus of the family *Flaviviridae* and genus *Flavivirus*. It has a positive-sense RNA genome encoding a polyprotein^1^. *Aedes aegypti* and *Ae*. *albopictus* are the most competent mosquito species for dengue transmission^1,2^. DENV infection in humans can cause diseases ranging from mild self-limited dengue fever (DF) to life-threatening dengue hemorrhagic fever (DHF) and dengue shock syndrome (DSS)^2,3^. Sequential infections by different serotypes of DENV normally result in more severe DHF and DSS due to the cross reaction of the host’s immune cells^4^. So far, DENV has prevalent in over 100 countries worldwide, and more than 100 million individuals are infected per year in the tropics and subtropics, including South America, Africa and Southeast Asia countries, where the competent vectors exist^3^. Dengue has become a global public health concern because of its fast geographic expansion and increased incidence worldwide, particularly in developing and undeveloped countries, where it has become a severe social and economy burden of local governments^5^. However, an approved DENV vaccine or anti-DENV drugs are still not available. The prevention and control of DENV outbreaks still depend on the effective vector control and protection of DENV-infected individuals by shielding strategies^6,7^.

South China is a hyper-endemic area of arboviruses, where *Aedes aegypti* and *Ae*. *albopictus* are widely distributed^8,9^. The outbreak of dengue was initially reported in 1978 in Guangdong, China^10^. In 2010, Chikungunya virus (CHIKV) caused an outbreak in Guangdong, China^11^. At present, all four serotypes of DENV are suspected to have been circulating in these areas since 1978^10^. Dengue outbreaks have been frequently reported in several provinces of South China, including Guangdong^12–16^, Guangxi^17^, Yunnan^18^, Fujian^19^ and Zhejiang^20,21^. In the past three decades, three large-scale dengue outbreaks occurred in 1980 (454,205 cases)^10^, 1986 (118,987 cases)^10^ and 2014 (45,236 cases)^22^. Particularly in 2014, multiple strategies were taken to prevent the expansion of DENV in Guangdong, including enhanced vector surveillance and prevention strategies, alerts about imported and autochthonous cases, protection of confirmed dengue cases in local hospital and announcements of traveler advice in Guangdong^22^. The knowledge gathered from the third large-scale dengue outbreak suggested that it resulted from a lack of sufficient tracing or alerting system of imported cases, less awareness of self-preventative measures against DENV infection, and untimely vector control measures^22^. Hence, at the beginning of 2015, the public health authorities of Guangdong launched the guideline of dengue prevention and laboratory diagnosis (2015–20). Unfortunately, multiple outbreaks of dengue occurred in 2015 in Guangdong, China, following the third historically large outbreak of dengue in 2014. The epidemiology, vector surveillance, control strategies and molecular characteristics of the DENV isolates involved in three cities (Guangzhou, Chaozhou and Foshan) were investigated to elucidate the potential gaps in the existing dengue prevention and control system of Guangdong, China.

## Methods

### Ethics statement

The serum sampling protocol was approved by the Ethics Committee of the Guangdong Provincial Center for Disease Control and Prevention (Guangdong CDC). All associated procedures were performed in accordance with the humanization regulations. Patients’ information was extracted from local hospitals. All subjects provided informed consent.

### Epidemiological data and serum sample collection

**Figure 1:**
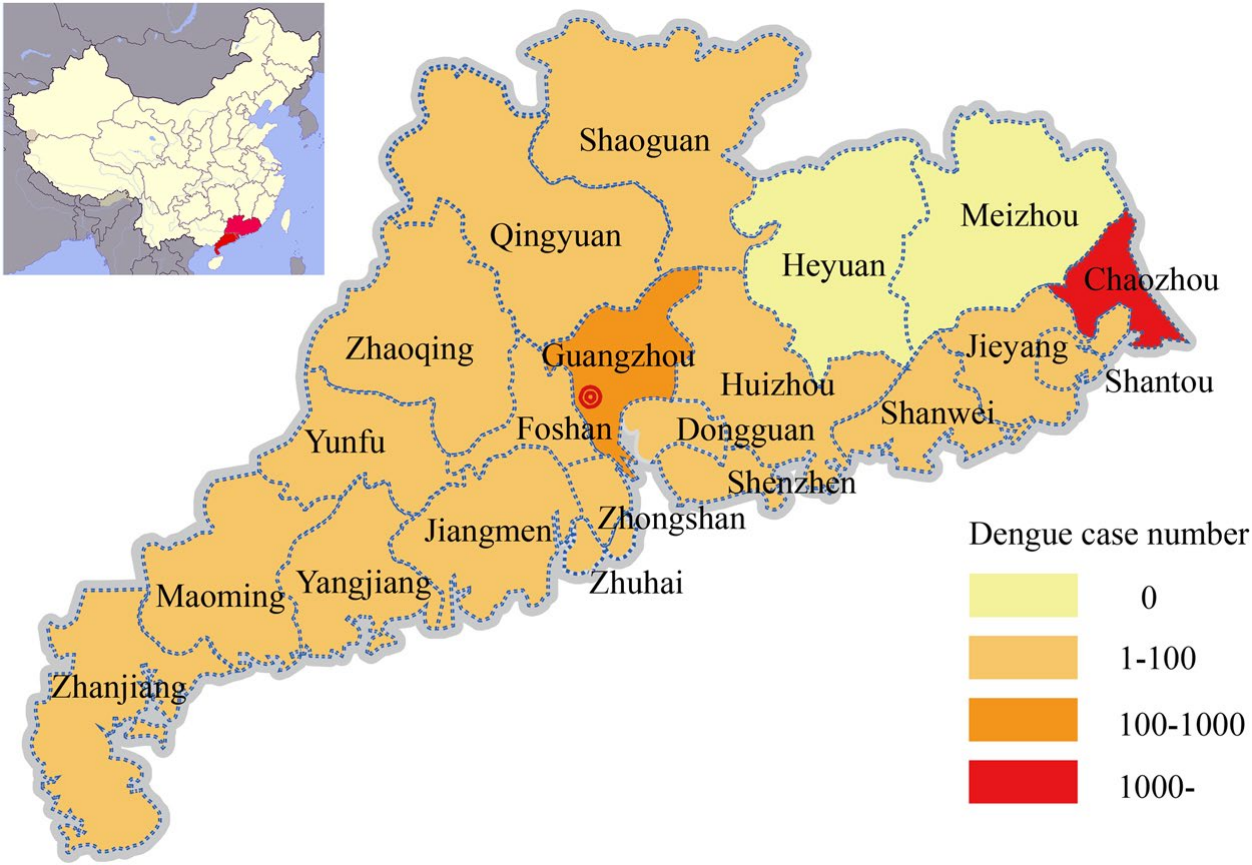
Cumulative incidence of dengue cases by parish during the 2015 outbreak in Guangdong, China (n = 1,699). The maps were plotted with ArcGIS 9.3 (ESRI) using the data from the Notifiable Infectious Disease Report System (NIDRS).

Guangdong located in south of China which closed to Southeast Asia countries (Fig. 1). Epidemiological data of dengue cases including the patient’s age, gender, address, profession, date of symptom onset, confirmation by laboratory test or not, origin (local or imported), and travel history were recorded by the local hospitals in 21 cities of Guangdong province. The data were uploaded to the Notifiable Infectious Disease Report System (NIDRS) by local hospitals. Duplicate records for the same cases were removed from the NIDRS. Serum samples were collected and delivered to the local CDC. Laboratory-confirmed samples were sent to the Guangdong CDC for dengue virus isolation and E gene sequencing.

### Case definitions

The cases were classified according to the diagnostic criteria for dengue fever (WS746-2014) of the Ministry of Health of the People’s Republic of China as previously reported^22^. Briefly as, suspected cases of dengue had more than two symptoms of acute onset fever, severe headache, orbital pain, myalgia, arthralgia, fatigue, flush, rash, and conjunctival congestion, and (1) with a history of travel in a dengue endemic area within 15 days before symptom onset, or cohabitation with an individual with confirmed dengue; or (2) a negative travel history, while with leucopenia or thrombocytopenia.

Suspected cases with leucopenia or thrombocytopenia and serum IgM positivity on the acute phase (within 7 days after symptom onset) were considered as clinically diagnosed cases. Laboratory-confirmed cases were those in which the patient was found to be positive for dengue virus RNA by real-time RT-PCR or for nonstructural protein 1(NS1) in acute-phase serum; those in which the virus was isolated from an acutely infected patient’s serum.

Imported and autochthonous dengue cases were defined according to the overseas travel history of the suspected patients, particularly whether they had been in a dengue endemic region within two weeks prior to their illness.

### Laboratory diagnosis and virus identification

Primary laboratory diagnosis was performed at the local hospital or local CDC. Patients were evaluated for the presence of IgM and NS1 using commercial ELISA kits (PanBio, Windsor, Australia). Universal real time RT-PCR was used to initially detect DENV in acute-phase serum specimens by serotype-specific real-time RT-PCR^23^. The real time RT-PCR positive samples were used for virus isolation as previously reported^22^. The supernatants of the positive samples were harvested and stored at −80 °C.

The envelope protein-coding gene (E gene) of dengue virus was amplified and sequenced for dengue virus identification. The total viral RNA was extracted from the cell culture supernatants using a QIAamp Viral RNA Mini Kit (Qiagen, Hilden, Germany)^24^. The E gene of DENV was amplified using a One-Step RT-PCR Kit (Qiagen, Germany), with the primers listed in Table 1 as previously reported. The PCR products were purified and sequenced using the same primers as those used in the PCR performed by Life Technology Company (Applied Biosystems, Shanghai, China). The obtained sequences were edited using SeqMan software from Lasergene software package (DNASTAR, Madison, USA). The similarity of the assembled sequences was searched for in the GenBank database (www.blast.ncbi.nlm.nih.gov/Blast.cgi).

**Table 1:** Primers used for E gene amplification.

DENV serotypes	Primer	Nucleotide sequences (5′-3′)
DENV-1	D1F1	CACATGCCATAGGAACATCCA
	D1F2	GGAACAGACAAGATTTGCTGGT
	D1R1	ATGAGCCTGTGCACATCACA
	D1R2	TGCCACTTCCACATTTGAGT
DENV-2	D2F1	TGGCATACACCATAGGAACGA
	D2F2	CCTCGACTTCAATGAGATGGT
	D2R1	CCTTTGAGCTGTAGTTTGTCCA
	D2R2	TTGAAGGGGATTCTGGTTGGA
DENV-3	D3F1	AGGGTTCACAATACTAGCCCTA
	D3F2	GTTCTCCATTCTGGTTGTCGA
	D3R1	GGGCTACAACAGAAACACCA
	D3R2	GTTCTCCATTCTGGTTGTCGA
DENV-4	D4F1	AGCTGGATACTCAGAAACCCAGGATT
	D4F2	CCTCATGCCAAGAGACAGGATGT
	D4R1	ACATCCTGTCTCTTGGCATGAGG
	D4R2	AATTTGTACTGTTCTGTCCAAGTGTG

### Phylogenetic analysis

An E gene data set (8391 sequences) of DENV-1 to −4 with clear origins and sampling data was initially selected by retrieving all of the DENV E gene sequences available in GenBank (DENV-1, 3449; DENV-2, 2645; DENV-3, 1540; DENV-4, 757). These sequences were first selected using the following strategy. One reference sequence was selected to represent the sequences that shared over 99% similarity detected in each country of each year. Then, the selected E gene sequences were aligned with 108 E gene sequences identified in isolates from the 2015 outbreak (DENV-1, 26; DENV-2, 61; DENV-3, 15; DENV-4, 6) using the Mega 6.06 program^25^. A maximum likelihood (ML) tree was then constructed using Mega 6.06, with 1000 bootstraps. The bootstrap-supported clusters (>80%), which comprised outbreak isolates in 2015 and closely related isolates in the same or neighboring clusters, were selected for further analysis. A total of 877 E endemic and sylvatic dengue virus E gene sequences (DENV-1, 373; DENV-2, 245; DENV-3, 157; DENV-4, 102) were selected as references for tracking the source of the 2015 outbreak isolates. The general time reversible (GTR) nucleotide substitution model with a proportion of invariant sites was identified as the best fitting model for ML inference by jModelTest v.1.6^26^. Maximum likelihood (ML) phylogenetic trees were inferred with Mega 6.06 using the General Time Reversible model and a gamma distribution (G + I, 4 nucleotide substitution model) with 1000 bootstraps.

### Phylodynamics of DENV-2

The temporal signal analysis of the correlation between the root-to-tip genetic divergence of DENV-2 and the date of sampling was conducted in TempEst^27^. The correlation between the sampling date of each sequence and the genetic distance of that sequence from the root of a maximum likelihood phylogeny of DENV-2 indicated that the sylvatic genotypes are the oldest representatives of DENV-2 that came from the environment or other hosts. They fall on the regression line, while extending the time of the common ancestor of DENV-2 (tMRCA = 1756.24, Slope = 7.56 × 10^−4^, R^2^ = 0.75) compared with the data set without this genotype (tMRCA = 1899.38, Slope = 6.55 × 10^−4^, R^2^ = 0.70). Thus, the data set including the sylvatic genotypes might not represent the evolution of the DENV-2 outbreak worldwide. Subsequent molecular clock phylogenetic analyses were therefore conducted using 284 E coding sequences without sylvatic genotypes. Recombination was assessed using GARD from the Data monkey software suite. This tool did not indicate the apparent recombination in this data set of DENV-2^28^.

Bayesian phylogenies were estimated in BEAST v.1.8.1^29^ using relaxed uncorrelated molecular clock (UCLN) models under the GTR nucleotide substitution model^30^. Three independent Markov chain Monte Carlo (MCM) runs of 100 million steps were computed, and 10% burn-in was discarded from each run. Convergence and behavior of the MCMC was inspected using Tracer v1.6 (http://beast.bio.ed.ac.uk/Tracer). The summary phylogenies were visualized in FigTree v.1.4.2 (http://tree.bio.ed.ac.uk/software).

### Entomological surveillance, control measures and climate characteristics

Guangdong is a hyper-endemic area of arboviruses. *Ae. albopictus* is the major competent vector in Guangdong, China, and widely are distributed throughout the province. *Ae*. *aegypti* are only distributed in south part of the province, and rarely countered during the dengue outbreak since 1978^10^. After 2014, a reinforced entomological surveillance was implemented throughout Guangdong provinces with increasing ovitrap numbers, increased surveillance frequency and advanced trap equipment. The risk threshold for *Ae. albopictus* density was interpreted by the Breteau index (BI) (number of containers with mosquito larvae per 100 premises/houses checked, with 10 > BI ≥ 5 indicating low risk, 20 > BI ≥ 10 denoting moderate risk, and BI ≥ 20 indicating high risk). In briefly, during the routine surveillance, 100 BI index data are collected each two weeks in each town with density equal to about 30–50 m^2^/BI index data. In case of dengue outbreak, three regions are classified around dengue case (Fig. S1). The first region is the core area which is a circle around the case with radius equal to 200 m. The second region is the warning area which is a ring around the core area with radius equal to 400 m. The third region is the monitoring area which is another ring around the warning area with radius equal to 600 m. all three regions can be combined or merged when there are multiple outbreak dengue cases. The BI index data are collected at the same density with the routine surveillance, while with higher frequency. The BI index data are collected within two days since outbreak reported in core regions, and repeated each three days until the BI < 5. In warning regions, BI index data are collected each one week until the BI < 5. In monitoring regions, these data are collected each two weeks until the end of this outbreak.

**Figure 2:**
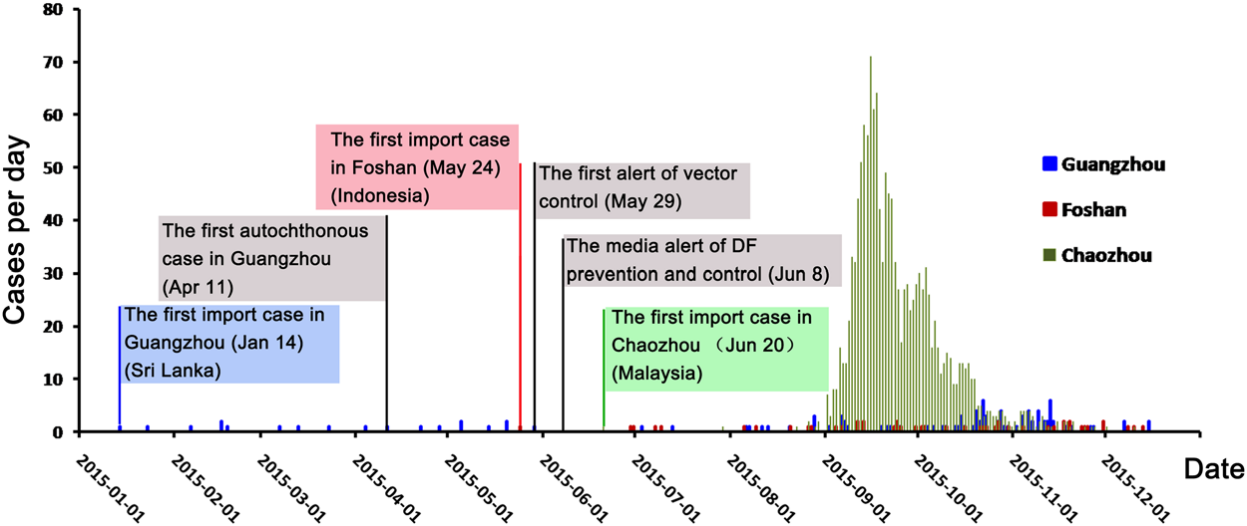
Reported dengue fever cases per day in Chaozhou, Guangzhou and Foshan cities, Guangdong, 2015 (n = 1,699). The colorful frame indicates the imported cases, with data on the nations visited by the travelers; the gray frame indicates the time course in which the control measures were implemented during this outbreak. The incidence of dengue cases were indicated with blue, red and green in Guangzhou, Foshan and Chaozhou, respectively.

The health authorities started the implementation of control measures at the beginning of 2015 (Fig. 2). In April of 2015, the first alert of entomological surveillance data was published by the Guangdong province health authorities (http://www.gdwst.gov.cn/a/zwxw/2015052913654.html). On May 7, a new guideline (2015–20) for dengue prevention and laboratory diagnosis was published by the Guangdong province health authorities. Six levels of emerging responses were defined according to the following guidelines: I, BI index >10 in one community or town, but no case reported; II, BI index >10 with imported or autochthonous cases; III, BI index >10, with autochthonous cases >5 within one week in one community or town; IV, BI index >10 with autochthonous cases >10, or >1-fold of average cases within 5 years in the same area; V, BI index >10 with autochthonous cases >100, or >2-fold of average cases within 5 years in the same area or incidence of IV events in at least two cities in Guangdong; and VI, incidence of V events in at least two provinces, including Guangdong. The first alerts on imported DENV cases were published by the public health authorities in January, May and June in Guangzhou, Foshan and Chaozhou, respectively (Fig. 2). On June 8, the media alert of dengue prevention and control was advertised. Control measures for mosquitos were implemented throughout Guangdong province until the end of this outbreak. Firstly, the public health authorities posted information on websites, traditional and social media to show the clinical appearance of dengue, stress the importance of mosquito clearance and personal protective measures, as well as patients protection in hospital. Secondly, the communities in the core, warning and monitoring regions started to eliminate the potential mosquitos breeding sites, including the cultivating water plants, waste tires and any other vessels in outdoors containing water. Thirdly, the local government started to kill mosquitoes by using pyrethroid mosquitocide including deltamethrin, cypermethrin and allethrin which all are WHO suggested. The clearance of mosquitoes were conducted each 3 days, and repeated 3–5 times until BI < 5 or without case report within 25 days.

The climate dataset including temperature, rainfall, and relative humidity was obtained from the China Meteorological Data Sharing Service System (http://cdc.nmic.cn/home.do). The plots of were performed using the “ggplot2” package in R 3.4.1.

## Results

### Epidemiological findings of the outbreak

During 2015, 1,699 suspected cases were reported in 19 cities in Guangdong. Of these, 1,627 were laboratory-confirmed cases, and 72 were clinically diagnosed cases (Table 2). All of the clinically diagnosed cases were suspected cases with leucopenia or thrombocytopenia and serum IgM positivity according to the diagnostic criteria for dengue fever (WS746-2014) enacted by the Ministry of Health of China. Among the 1,627 laboratory-confirmed cases, 1,194 (73.38%) were verified by real-time RT-PCR (44, 1126, 18 and 6 for DENV-1, -2, -3 and -4, respectively), while 433 (26.62%) were established by NS1 ELISA. A total of 1,194 serum samples from 19 cities were submitted to the Guangdong CDC. All of the serum samples that tested positive for the viral RNA or NS1 were accepted by the Guangdong CDC and used for virus isolation. A total of 312 dengue virus isolates were successfully obtained, including 44 DENV-1, 161 DENV-2, 15 DENV-3 and 6 DENV-4 isolates. Twenty-six and sixty-one isolates were randomly selected as representative of DENV-1 and -2, respectively (five representative isolates were selected if the total isolates number was >5, and all isolates were selected if the total isolates number was ≤5 in the same outbreak locations), while all of the DENV-3 to -4 isolates were sent for E gene sequencing, resulting in 108 E gene sequences (DENV-1, 26; DENV-2, 61; DENV-3, 15; DENV-4, 6) (Fig. S2).

**Table 2:** Summary of DENV infection cases and distribution in Guangdong, 2015.

	All cases	Lab confirmed cases	Clinic identified cases	China Mainland	China Hongkong & Marco	Foreigner
Chaozhou	1382	1360	22	1382	0	0
Guangzhou	111	85	26	101	0	10
Shenzhen	47	42	5	46	0	1
Foshan	47	43	4	45	0	2
Shantou	22	13	9	22	0	0
Zhongshan	22	20	2	22	0	0
Dongguan	21	19	2	21	0	0
Yangjiang	11	10	1	11	0	0
Jiangmen	8	8	0	8	0	0
Jieyang	7	7	0	7	0	0
Shaoguan	6	6	0	5	0	1
Zhanjiang	3	3	0	3	0	0
Maoming	3	3	0	3	0	0
Huizhou	3	3	0	2	1	0
Zhuhai	2	2	0	2	0	0
Zhaoqing	1	1	0	1	0	0
Shanwei	1	1	0	1	0	0
Qingyuan	1	1	0	1	0	0
Yunfo	1	0	1	1	0	0
Meizhou	0	0	0	0	0	0
Heyuan	0	0	0	0	0	0
Total	1699	1627	72	1684	1	14

Forty-five imported cases were confirmed to have originated in Southeast Asia (Table 3). The daily time course of the recorded dengue fever cases was shown in Fig. 2. Of the total cases, 844 (49.67%) occurred in males and 855 (50.33%) occurred in females. The median age was 41 years (range 5–65). The most affected age group was the 20- to 60-year-old group. No fatalities were recorded. Unemployed individuals (661, 38.91%), retirees (218, 12.84%), retailers (98, 5.76%), workers (225, 13.24%), students (125, 7.35%) and farmers (63, 3.72%) were the most affected populations (Table 4). The highest proportion of patients was from Chaozhou (1382, 81.34%), Guangzhou (110, 6.47%), Shenzhen (47, 2.77%) and Foshan (47, 2.77%), according to the current geographic distribution of cumulative dengue cases by parish of residence (Fig. 1).

**Table 3:** Summary of imported DENV infection cases and serotype distribution in Guangdong, 2015.

Countries	DENV-1	DENV-2	DENV-3	DENV-4	Total
India	0	3	0	0	3
Vietnam	1	1	0	0	2
Indonesia	2	2	3	0	7
Sri Lanka	3	0	0	0	3
Myanmar	1	2	0	0	3
Bangladesh	2	0	0	0	2
Thailand	1	1	1	1	4
Malaysia	5	1	1	0	7
Maldives	0	2	0	0	2
Cambodia	0	1	0	3	4
Philippines	1	0	3	2	6
Brazil	1	0	0	0	1
Papua New Guinea	0	1	0	0	1
Total	17	14	8	6	45

**Table 4:** Dengue fever cases by profession, Guangdong, China (n = 1,699).

Profession	cases	Proportion
Unemployed	661	38.91%
Retiree	218	12.84%
Trader, Retailer	98	5.76%
Worker	225	13.24%
Student	125	7.35%
Farmer	63	3.72%
Unknown	47	2.76%
Officer	55	3.23%
Child (at home)	20	1.17%
Teacher	23	1.35%
Child (in kindergarten)	7	0.42%
Doctor	12	0.71%
Cook, Waiter	17	1.01%
Fisherman	1	0.06%
Others	127	7.47%
Total	1699	100%

### Phylogenetic analysis

The E protein region was sequenced from 108 isolates, including 26 DENV-1, 61 DENV-2 and 15 DENV-3 and 6 DENV-4 isolates. A total of 877 E gene reference sequences (DENV-1, 373; DENV-2, 245; DENV-3, 157; DENV-4, 102) were selected from GenBank in initial phylogenetic analysis for original tracking of the 2015 dengue outbreak. Phylogenetic analysis for each dengue virus serotype showed that multiple resources likely contributed to this outbreak.

**Figure 3:**
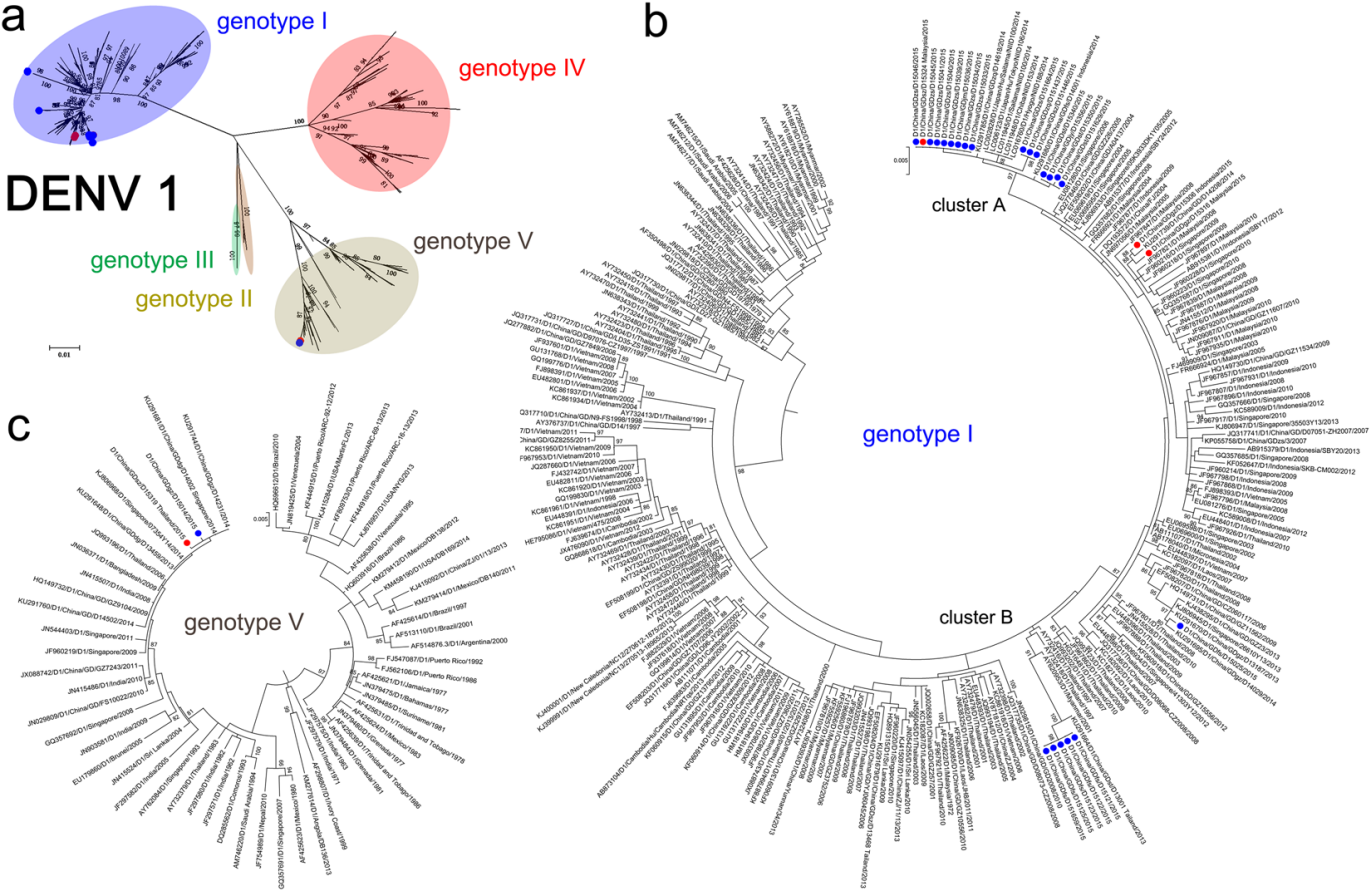
Phylogenetic tree of DENV-1 isolated from the 2015 outbreak in Guangdong, China. Twenty-six E gene sequences from isolated strains and 373 reference sequences from GenBank were used for phylogenetic tree reconstructions. Five genotypes were assigned according to previous studies (**a**). The outbreak isolates are indicated by blue circles, and the isolates from the imported cases are indicated by red circles. Two clusters (A and B) were assigned to genotype I of DENV-1 (**b**); two extra isolates were assigned to genotype V(c).

Twenty-one DENV-1 isolates were assigned to two independent clusters (A and B) in genotype I (Fig. 3). The other five DENV-1 isolates (GDgz/D15306, GDgz/D15014, GDgz/D15316, GDgz/D15319 and GDfs/D15025) were separated in genotypes I and V, of which GDgz/D15306, GDgz/D15316 and GDgz/D15319 were imported cases from Indonesia, Malaysia and Thailand, respectively. In particular, GDfs/D15025 was clustered with other strains previously detected in China. Genotype I is the most endemic genotype of DENV-1 worldwide. Cluster A comprised 16 outbreak isolates from 6 cities in Guangdong, and it was clustered together with isolates imported from Malaysia. The recent ancestors of this cluster were the isolates that had circulated in Thailand in 2008. The isolates of Cluster B (5 isolates, Foshan) were highly similar to those obtained in Thailand in 2013.

**Figure 4:**
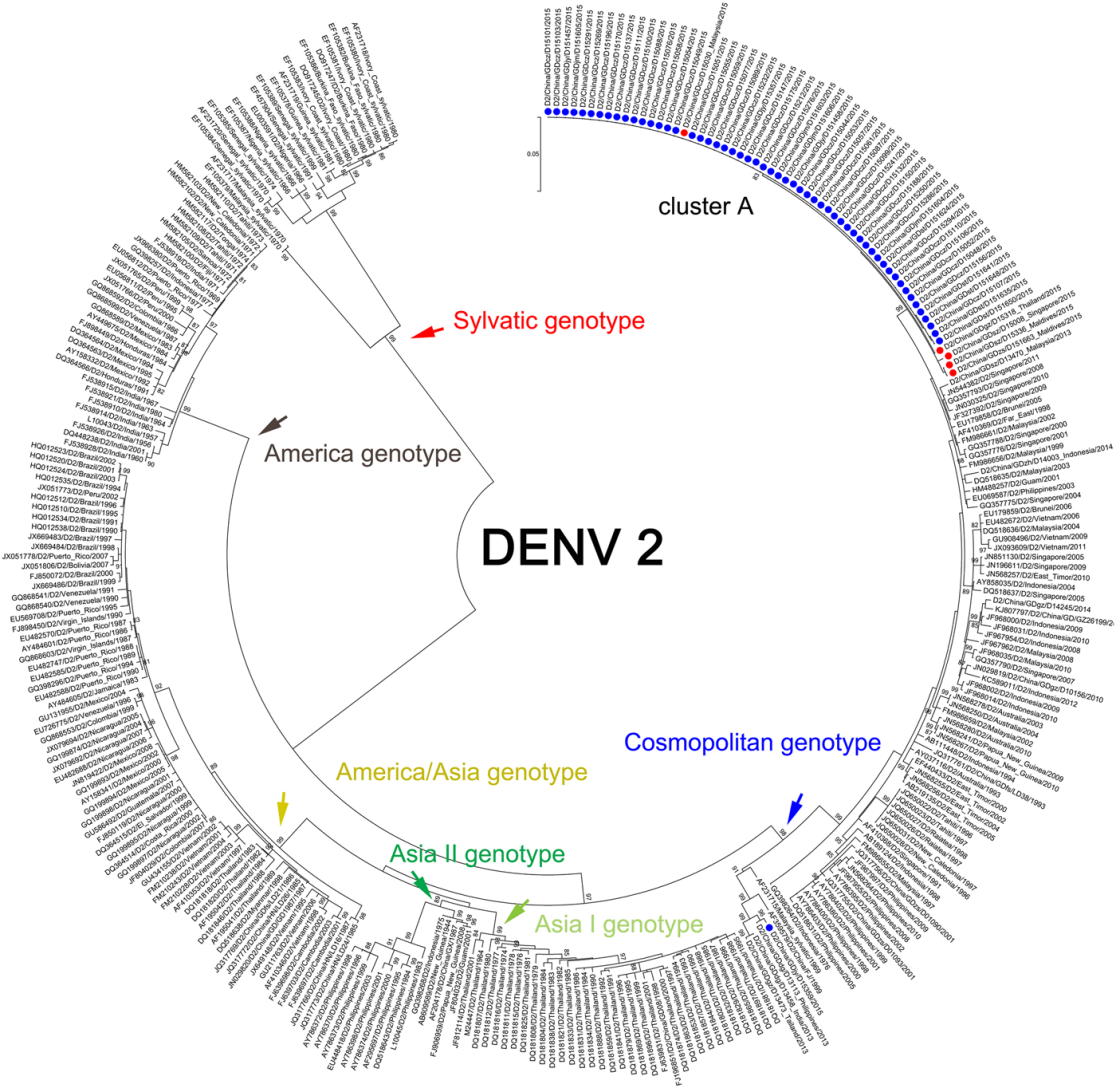
Phylogenetic tree of DENV-2 isolated from the 2015 outbreak in Guangdong, China. Sixty-one E gene sequences from isolated strains and 245 reference sequences from GenBank were used for the phylogenetic tree reconstructions. Six genotypes were assigned according to previous studies. One cluster (n = 60) was assigned to the Cosmopolitan genotype of DENV-2. The extra isolates also belonged to this genotype but were not clustered together with the other sixty isolates. The outbreak isolates are indicated by blue circles, and the isolates from the imported cases are indicated by red circles.

DENV-2 is the major outbreak serotype of Guangdong, China, in 2015. A total of 1382 DENV-2 infection cases were identified, of which 61 DENV-2 strains were selected for E gene sequencing. All DENV-2 outbreak isolates were assigned into single cluster A belonging to the Cosmopolitan genotype (Fig. 4). The most recent origins of cluster A (60 isolates, 4 cities) were the imported isolates that were endemic in Malaysia, Thailand and the Maldives in 2015. The extra single isolate GDjy/D15359 was reported as an autochthonous case, and its origin remained unknown. Surprisingly, we found that it could be clustered with imported DENV-2 isolates in Dongguan and Guangzhou imported from the Philippines, Thailand and India in 2013.

**Figure 5:**
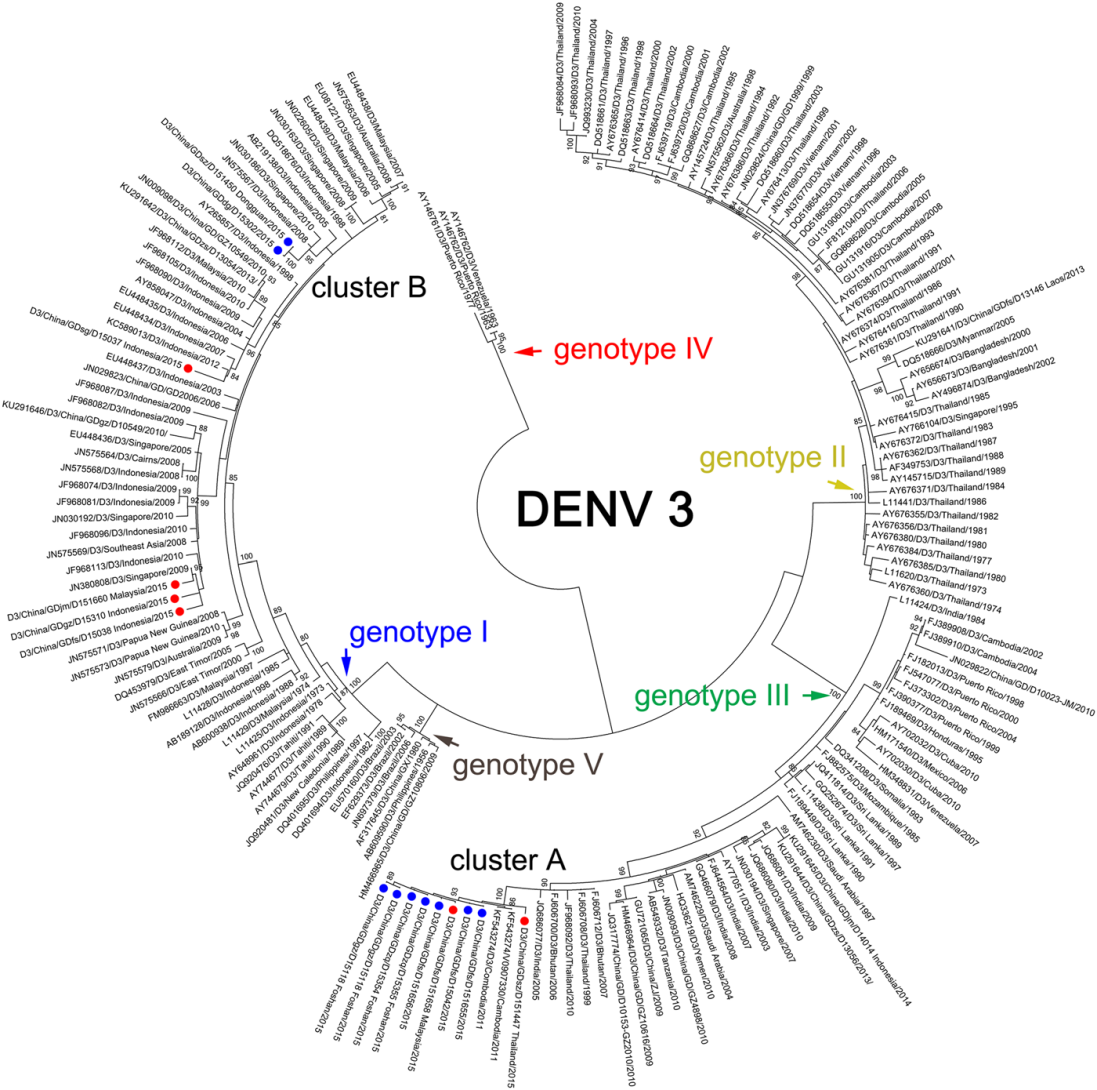
Phylogenetic tree of DENV-3 from the 2015 outbreak in Guangdong, China. E gene sequences from 15 isolates and 157 reference sequences from GenBank were used for phylogenetic tree reconstructions. Five genotypes were assigned according to previous studies and are highlighted in different colors. The outbreak isolates are indicated by blue circles and are divided into two separate clusters. The imported cases are indicated by red circles.

Nine autochthonous and six imported DENV-3 infection cases were identified in Guangdong, China, in 2015, and these were assigned to two clusters (Fig. 5). Cluster A belonged to Genotype III and comprised seven autochthonous cases reported in three cities (Guangzhou, Zhaoqing and Foshan) and two imported cases from Thailand and Malaysia. Cluster B belonged to Genotype I, but there was no index case in this cluster. The most recent source of these two isolates was likely from Indonesia and Singapore between 2005 and 2010. The extra four cases were imported from Indonesia and Malaysia.

**Figure 6:**
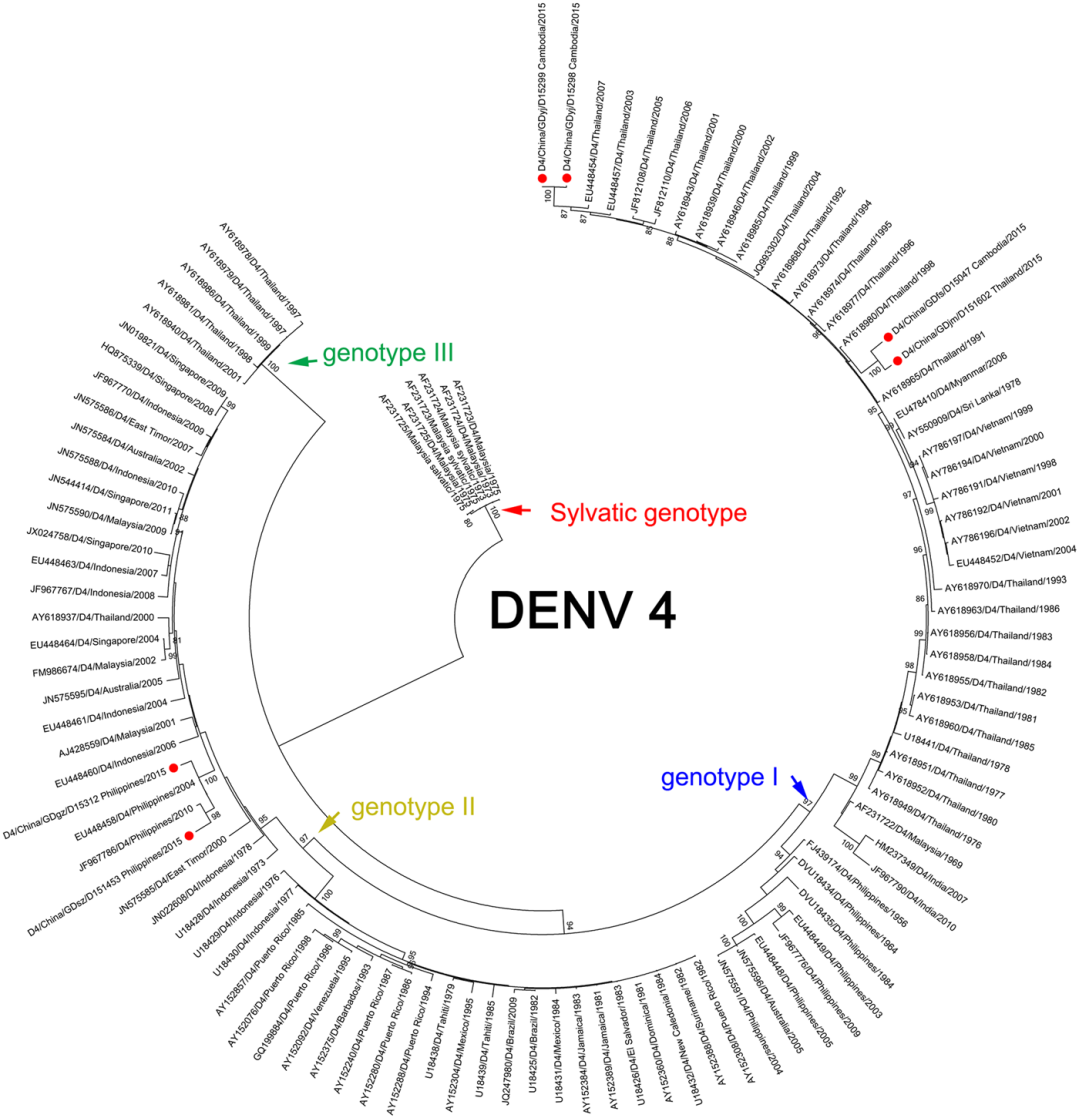
Phylogenetic tree of DENV-3 from the 2015 outbreak in Guangdong, China. E gene sequences from 15 isolates and 157 reference sequences from GenBank were used for phylogenetic tree reconstructions. Five genotypes were assigned according to previous studies and are highlighted in different colors. The outbreak isolates are indicated by blue circles and are divided into two separate clusters. The imported cases are indicated by the red circles.

All DENV-4 outbreak strains were isolated from imported cases from individuals who travelled in Cambodia, Thailand and the Philippines (Fig. 6). No DENV-4 infected autochthonous cases were reported.

### Phylodynamics of DENV-2

**Figure 7:**
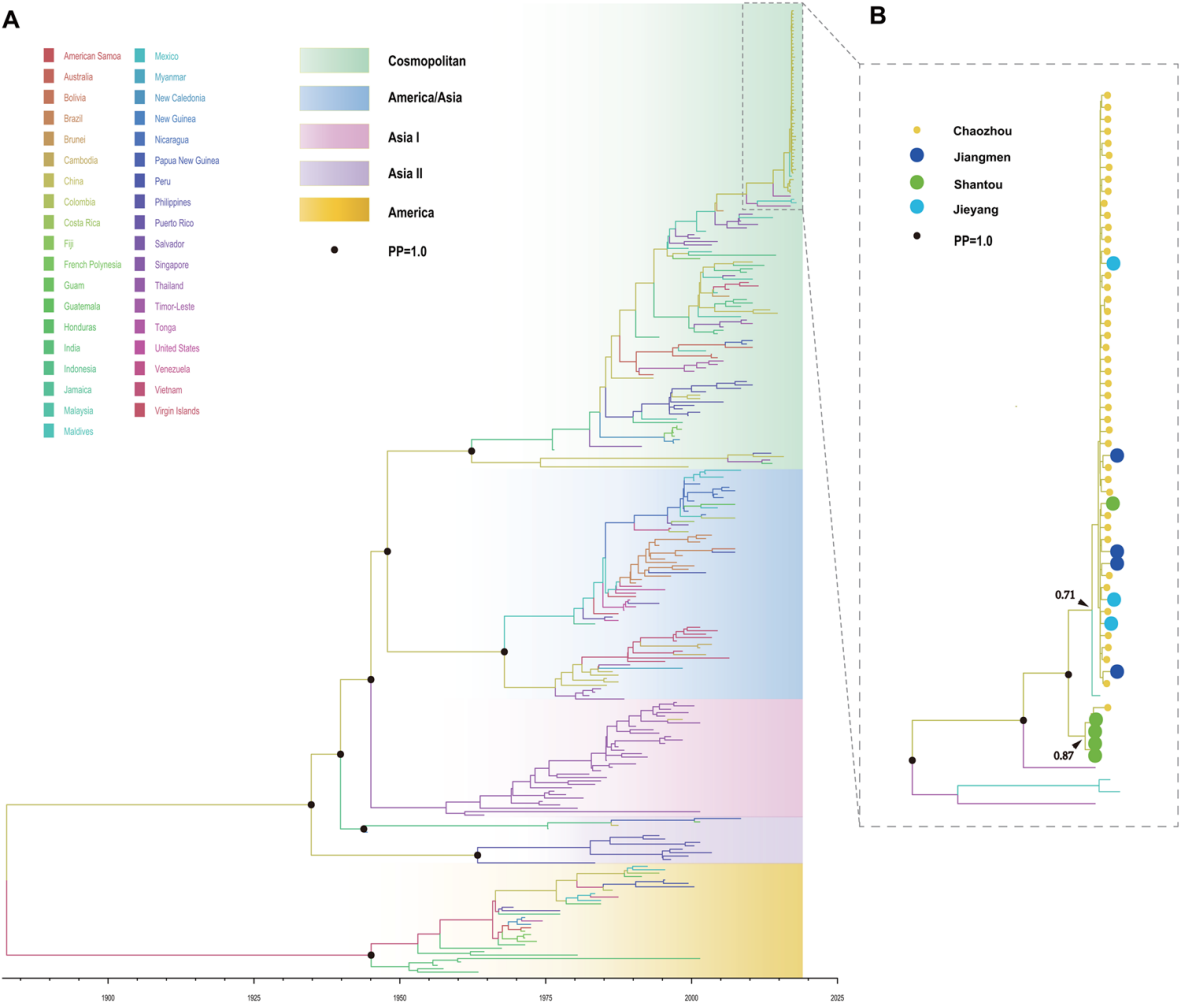
Maximum clade credibility tree from the Bayesian analysis of DENV-2. A maximum clade credibility (MCC) tree was constructed using the BEAST program. Each genotype is marked with different colors. The black circle indicates the posterior probability (PP = 1.0) value (**A**). The outbreak branch in Chaozhou was expanded in (**B**), and the original source of each isolate is marked in different colors. The black circle indicates the posterior probability (PP = 1.0) value.

Since DENV-2 was the major serotype during the outbreak, 61 DENV E genes were sent for further phylodynamic analysis combined with a global set E gene of DENV-2 without sylvatic isolates. Phylodynamic analysis of 284 E gene sequences showed that all DENV-2 sequences were assigned to one of five genotypes: the Asia I, Asia II, America/Asia, Cosmopolitan, or America genotype. The estimated evolutionary rate of DENV-2 under the selected evolutionary model was 6.51 × 10^−4^ (95% HPD: 5.91 × 10^−4^–7.11 × 10^−4^) s/s/y (Fig. 7A). The corresponding estimate of the time of the most recent common ancestors (tMRCAs) of DENV-2 was 1882 (95% HPD: 1880–1910), which was consistent with what has been previously reported^31–33^. The tMRCA of the Cosmopolitan genotype was 1962 (95% HPD: 1960–1963) (Fig. 7A).

These outbreak isolates of DENV-2 were clustered together except for the single isolate GDjy/D15359. Unlike the phylogenetic analysis, the time scaled phylodynamic analysis identified single cluster (Fig. 7B. Posterior Probability, PP = 1.0) during the outbreak, while revealed the extensive transmission of DENV-2 from Chaozhou to the Shantou and Jieyang, as well as the distant Jiangmen neighborhood (500 kilometers away) (Fig. 7B.). In addition, we identified two separate sub-clusters with low posterior probability support (PP = 0.71 and 0.87), which indicated two independently outbreaks of DENV-2 in Chaozhou and Shantou, respectively. The estimate of tMRCA of Chaozhou cluster was May, 2015 (95% HPD: February, 2015–Januray, 2016). The most common ancestor of this cluster was likely from Malaysia in 2015. The tMRCA of Shantou cluster was April, 2015 (95% HPD: October, 2014–April, 2016). The potential ancestor of this cluster was remained unknown.

### Entomological surveillance and control measures and climate characteristics

**Figure 8:**
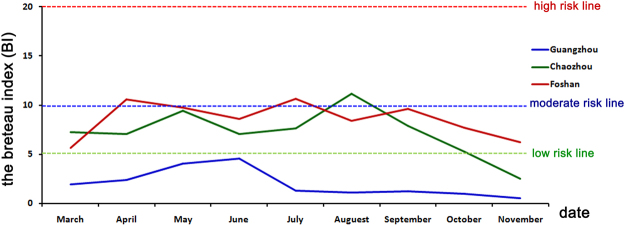
The results of enhanced surveillance of vectors in Chaozhou, Guangzhou and Foshan during the 2015 dengue outbreak in Guangdong, China. The Breteau index (BI) shows the density of *Ae. albopictus* in a block plus the surrounding block within a radius of 100 meters. BI < 5, safe; 10 > BI ≥ 5, low risk; 20 > BI ≥ 10, moderate risk; and BI ≥ 20, high risk.

**Figure 9:**
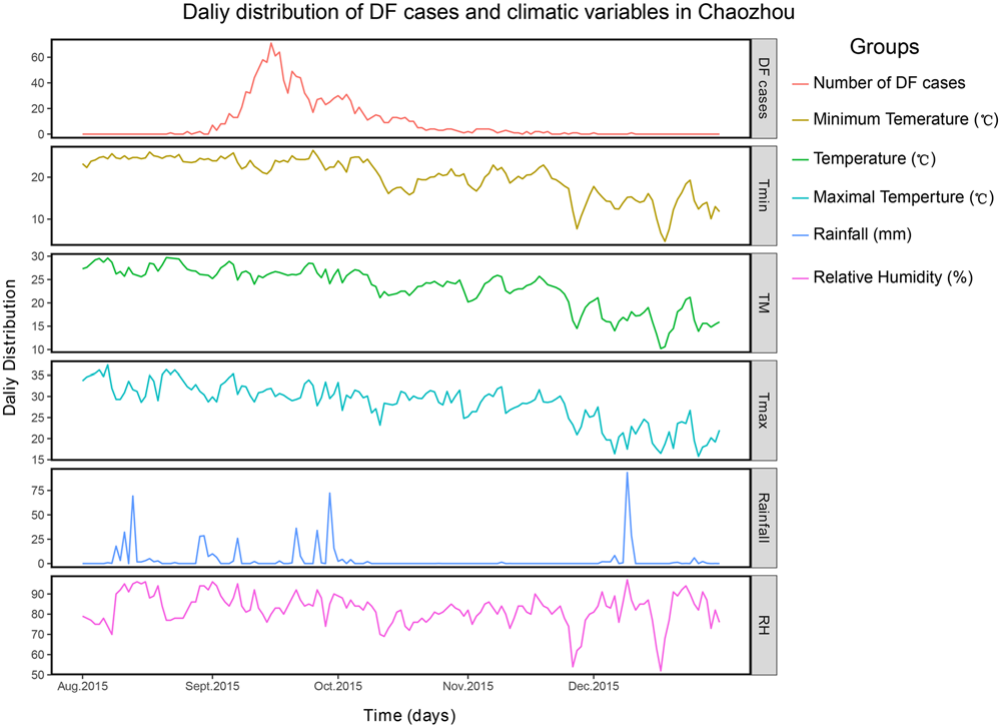
The climate tendency (temperature, rainfall, and relative humidity) and incidence of dengue cases in Chaozhou. The plots of were performed using the “ggplot2” package in R 3.4.1.

The enhanced surveillance systems in 2015 showed that the mosquito population was not reaching the upper risk threshold for *Ae. albopictus* density according to the BI in Chaozhou, Guangzhou and Foshan (Fig. 8). Particularly, for Guangzhou, the BI was kept at a low level from March to November. After control measures were undertaken continuously by the health authorities of Guangdong province, the BI decreased to the lower risk threshold at the end of September and November in all three cities (Fig. 8). The reported number of cases was significantly decreased at the middle of September in all three cities (Fig. 2). However, we found that the environmental factors associated with mosquitos’ activities, including temperature, rainfall and relative humidity (Fig. 9). There were no apparently difference of temperature during September to October (>25 °C) in Chaozhou. However, the rainfall were much more frequently during August to October, and drop down at the beginning of October. The average humidity changed frequently during the outbreak (80–90%), and drop down to 70–80% at the beginning of November.

## Discussion

Guangdong, China, is a hyper-endemic area of arboviruses, where both *Aedes aegypti* and *Ae*. *albopictus* are widely distributed^10,34^. The outbreak of dengue was initially reported in 1978 in Guangdong, China^10^. Thus far, all four serotypes of this virus have been encountered in this area since 1978^34^. Three large-scale dengue outbreaks occurred in Guangdong in 1980, 1986 and 2014^10,22^. In 2010, imported Chikungunya virus caused an outbreak in a single city of Guangdong, China, but no continuous circulation of the virus was observed after the outbreak^11^. In 2016, 18 imported Zika virus-infected cases were identified in a particular area of Guangdong, indicating that the returning of ex-patriot Chinese increased the risk for the local transmission of Zika virus^35–37^. Thus, Guangdong is in the front line of defensing arbovirus outbreak in China. The local public authorities issued control strategies refer to the prevention and control of potential outbreaks caused by arboviruses, including DENV, ZIKV and CHIKV. Unexpectedly, following a third historical outbreak in 2014, Guangdong faced its multiple outbreak of dengue occurring in 2015. The epidemiological, vector surveillance, control strategies and molecular characteristics of the DENV isolates in Guangzhou, Chaozhou and Foshan were investigated to elucidate potential gaps in the strategies for dengue prevention and control in Guangdong, China.

The major outbreak of dengue in Guangdong occurred in Chaozhou, and circulated in these areas from September to November of 2015. Four DENV serotypes were identified during the outbreak. DENV-2 was the major causative agent of the outbreak, and it accounted for 81.34% of all cases. Meta-analysis based on global data of 174 outbreaks during 1990 to 2015 reported that most monoinfection outbreaks were caused by DENV-2 (36, 20.7%)^38^. Between 1990 and 1994, DENV-2 was the most frequently identified serotype. It was also the most common serotype during 1995–1999 and 2000–2004^38^. In China, DENV-2 was the predominant serotype from 1986 to 1988 and caused the second largest dengue outbreak in Guangdong, Guangxi and Hainan provinces^10^. After 1999, DENV-2 was surpassed by DENV-1, and the genotype of DENV-2 also switched from an Asian/American to a Cosmopolitan genotype^10^. In this study, 60 DENV-2 cases were assigned to the Cosmopolitan genotype, while index cases were imported from Malaysia, Thailand and the Maldives. The phylodynamic studies of DENV-2 confirmed the results of the phylogeny analysis, indicating that this outbreak was caused by imported cases according to the time scale of those isolates shown in Fig. 7A. In addition, the phylodynamic results revealed the internal transmission of DENV-2 in Guangdong province, which suggested that this tool could be used for further transmission chain studies during the outbreak of dengue, as in recent implementation for Zika virus^39–41^ and Ebola virus^42^ monitoring.

DENV-1 was the second most identified serotype during this outbreak. Twenty-six DENV-1 isolates were assigned to genotypes I and V in this study. The most common ancestor of those DENV-1 isolates likely originated from Thailand. DENV-1 was first identified in Guangdong in 1999 and was found to be a unique serotype between 2001 and 2008. After 2010, although DENV-1 to -4 have been founded, DENV-1 became the predominant serotype of the DENV endemic in Guangdong^10,43^, and frequent genotype shifting from 2010 to 2014 depended on the original source of imported index cases, e.g., the outbreak genotype of DENV-1 switched frequently between genotypes I and IV from 2000 to 2005, and genotype I predominated from 2006 to 2013. In 2014, genotype V caused a large-scale outbreak of dengue in Guangdong, China^22^. DENV-3 was the major serotype from 1979 to 1982, and it caused the first large outbreak of dengue in three neighboring provinces, including the Guangdong, Guangxi and Hainan provinces^10^. It caused a larger outbreak in Guangzhou from 2009 to 2010^44^. During this outbreak, nine autochthonous and six imported DENV-3 infection cases were identified and assigned into two clusters with different geographical origins. Although available studies indicated that the DENV-3 (particularly genotype III) is easily transmitted^45^, we did not detect extra autochthonous DENV-3 infection cases during this outbreak. DENV-4 is considered to be positively correlated to severe disease risk, especially for children after secondary infection following antibody-dependent enhancement (ADE)^46^. In Thailand, DENV-4 accounted for 10% of DHF cases in children, and most DENV-4 DHF cases were associated with secondary dengue viral infection^46^. The endemic area of DENV-4 was widespread in American and Asian countries, including Thailand^46^, the Caribbean^47^, Brazil in 2007^48,49^, Easter Island^50^, Malaysia^51^ and India in 2009^52^. However, only a few outbreaks of dengue were caused by imported DENV-4 in Guangdong, China, e.g., in 1978, 1989, 2010 and 2012^15,17,53^. During 2015, six DENV-4 outbreak strains were isolated from imported cases of individuals who travelled from Cambodia and the Philippines, while no autochthonous cases were reported.

Referring to the epidemiological characteristics and phylogenetic analysis results, the 2015 dengue outbreak was likely caused by imported cases transmitted by *Ae. albopictus* due to fact that *Ae. aegypti* was rarely encountered during the dengue outbreak. The source of infection may have been the countries in Southeast Asia, such as Malaysia, Indonesia or Thailand, based on the phylogenetic analysis results and travel histories of the imported cases. However, a few autochthonous cases were clustered in independent clades that were linked to historical outbreak isolates in China. We are unsure of the relationship between these cases and potential unidentified imported cases or local circulated clades of DENV in Guangdong, which was reported in archived information from 2002, 2006, and 2014^12,22,54,55^. Although few researchers stated that they found continuously local circulated clades, dengue is still considered to be an imported disease in Guangdong, China^55–57^. Because most of the outbreaks in Guangdong were caused by imported index cases but not suspected local circulated clades of DENV. Another explanation is the lack of representative DENV isolates from Southeast Asian countries in the suspected local transmission clades due to limited epidemiological information on dengue virus infections in those countries (Malaysia, Singapore, Indonesia and Thailand), which served as major source of imported cases in China. Thus, it is difficult to draw any conclusions regarding local circulation for the independent clades of dengue in Guangdong, China.

Although the local public health authorities launched rigorous guidelines for dengue prevention and laboratory diagnosis (2015–20) at the beginning of 2015, the unexpected multiple outbreak of dengue occurred in Guangdong, China, particularly in Chaozhou where the outbreak cases accounted for 81.34% of all cases. The following reasons were considered to have prompted this outbreak of dengue: (I) There was a lack of sufficient tracing system and protection strategies of imported cases in Chaozhou. Indeed, the first alert regarding imported cases in Guangzhou, Foshan and Chaozhou was on January 10, May, 24 and June 20, respectively. The public health authorities launched an alert on dengue prevention and control on June 20. Five imported dengue cases were imported to Chaozhou as the index cases of this outbreak; thus, the protection of imported, acute-phase cases at local hospitals is a potential method for early outbreak control and prevention. (II) The vector control measures were not taken on time. Before the first alert of imported cases in Chaozhou, the public health authorities launched an alert of dengue prevention and control on June 20. Apparently, no vector control measures were taken before the following outbreak. In contrast, the vector control measures were implementation on time following an alert issued by the public health authorities in Guangzhou and Foshan. Consequently, no large outbreaks were detected in Guangzhou and Foshan, although there were more imported cases early in the year and with comparative BI levels compared with Chaozhou. (III)Timely screening tests of suspected cases will need to launch early-stage prevention programs after the local alert of the imported cases. On June 20, the first alert of imported cases was reported in Chaozhou. However, there is no associated surveillance regarding imported cases until September. (IV) Enhanced vector surveillance systems are needed in Chaozhou. A high risk of dengue outbreak is associated with a BI that exceeds 5^58^. The BI from April to August in Chaozhou and Foshan reached the moderate risk threshold. However, there were few ovitraps in Chaozhou, and the number was insufficient for identifying trends in the vector expansion. (V) A lack of awareness of self-preventative measures against dengue greatly contributed to its transmission. Chaozhou is located in the east of Guangdong. There have been few reports on dengue outbreaks in this city since 1978. Many residents in the community have had no experience with dengue, resulting in untimely hospitalization and failed attempts in mosquito prevention and extermination.

The number of reported cases significantly decreased at the middle of September; thus, the outbreak was under control at this time, and future cases could be prevented. The variance climate conditions during the outbreak of dengue in Chaozhou favored for mosquitoes breeding, and promoted the onward transmission of dengue virus in this areas, while those climate factors changed after September, it definitely hindered the breeding and activities of mosquitoes, and attributed to the drop down of dengue incidence in Chaozhou. However, the daily distribution of dengue cases indicated that the incidence of dengue virus infection was drop down from the middle of September, which suggest the case and vector control intervention strategies probably block the continuously transmission, and result in a break point of dengue cases distribution plot at the middle of September. Indeed, the total cases of this outbreak will reach to 8200 if without any case or vector control interventions though compartmental dynamic SEIR (Susceptible, Exposed, Infected and Removed) model^59^. In addition, the magnitude of the outbreak were highly sensitive to the intensity and timing of interventions, and showed as more rigorous, earlier the control interventions implemented, and more effective it yielded. In extreme cases, greatly impact on the prevalence and duration of dengue outbreak even if those control strategies were initiated several weeks after the onset of the outbreak. Thus, it suggested that implementation of dengue interventions were effective strategies for control and prevention of the outbreak of dengue in Chaozhou, 2015^59^. Therefore, it can be concluded that both the implemented control measures and variance of the natural environmental factors pose effects on the activity of mosquitoes (Fig. 9). The BI index was dramatically decreased after the control strategies were initiated in September. The future control measures should be improved and focused on reinforcing strategies involving the monitoring of all imported cases, implementing control policies in a timely way, and increasing the performance of vector surveillance. Moreover, the Guangdong CDC is a WHO-collaborating center in the West Pacific regions. The establishment of a potential cooperative international system with the endemic countries in Southeast Asia would certainly contribute to the control and prevention of dengue in Guangdong, as well as in West Pacific areas.

In conclusion, we have presented the epidemiological findings and phylogenetical characteristics of DENV isolates obtained during the outbreak in Guangdong, China. Multiple geographic origins likely contributed to this outbreak. The cosmopolitan type of DENV-2 was the major outbreak genotype. The comprehensive comparative studies within those three cities indicated the extreme importance of early detection, case management of imported cases, the ability to launch the suspected case surveillance following imported cases, the early implementation of control policies issued by local public health authorities, and the ability to reinforce the vector surveillance strategies for the further prevention of dengue epidemics in Guangdong, China.

## Electronic supplementary material

Supplementary Data
