# Supplementary material for: The epidemiological characteristics and molecular phylogeny of the dengue virus in Guangdong, China, 2015
Source: Sci Rep. 2018 Jul 2;8:9976. doi: 10.1038/s41598-018-28349-2 (PMC6028473; doi:10.1038/s41598-018-28349-2)
Supplement: Supplementary file 1 — Supplementary Data [file 41598_2018_28349_MOESM1_ESM.pdf]

Journal: Scientific Reports; MSID: SREP-17-46716B

The epidemiological characteristics and molecular phylogeny of the dengue virus in Guangdong, China, 2015

Running title: The molecular epidemiology of DENV in Guangdong, China, 2015.

Jiufeng Sun[1], Huan Zhang[2,3], Qiqi Tan[2,3], Huiqiong Zhou[2,3], Dawei Guan[2,3], Xin Zhang[2,3], Jinhua Duan[2,3], Songwu Cai[2,3], Zhiqiang Peng[2,3], Jianfeng He[2,3], Changwen Ke[2,3], Jinyan Lin[2,3], Tao Liu[1], Wenjun Ma[1], De Wu[2,3#]

[1]*Guangdong Provincial Institute of Public Health, Guangdong Provincial Center for Disease Control and Prevention, Guangzhou, 511430, China.*

[2]*Key Laboratory for Repository and Application of Pathogenic Microbiology, Research Center for Pathogens Detection Technology of Emerging Infectious Diseases, Guangdong Provincial Center for Disease Control and Prevention, Guangzhou, 511430, China.*

[3]*WHO Collaborating Centre for Surveillance, Research and Training of Emerging Infectious Diseases. Guangzhou, 511430, China.*

Corresponding author: De Wu, Institute of Microbiology, Center for Disease Control and Prevention of Guangdong Province, Guangzhou 511430, China.

Tel.:+86(020)31051091; E-mail: wude68@tom.com

#

#

#
#

Supplementary Figure legends:

Fig. S1.regions classified around dengue cases in case of dengue outbreak.

Fig. S2. A flowchart of reported dengue cases during 2015, Guangdong, China.

#

Regions classified around cases in case of dengue outbreak

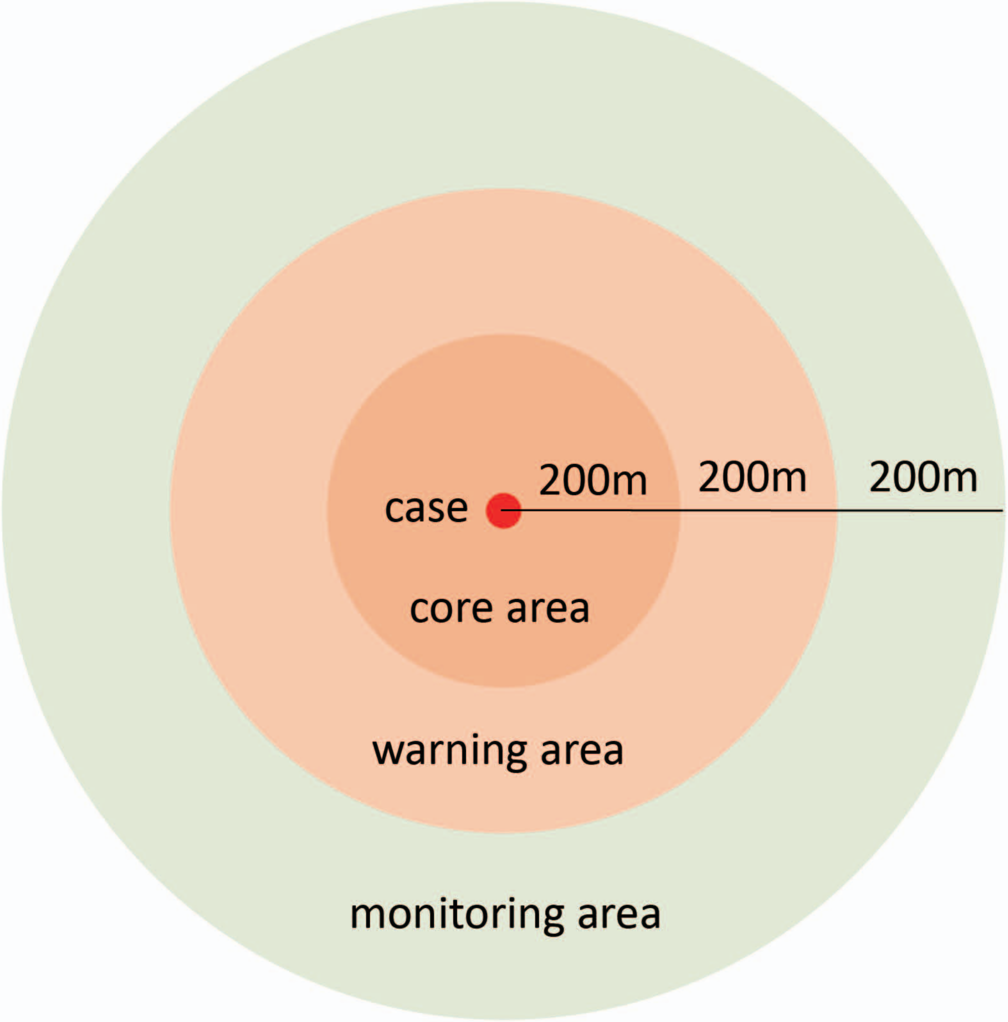

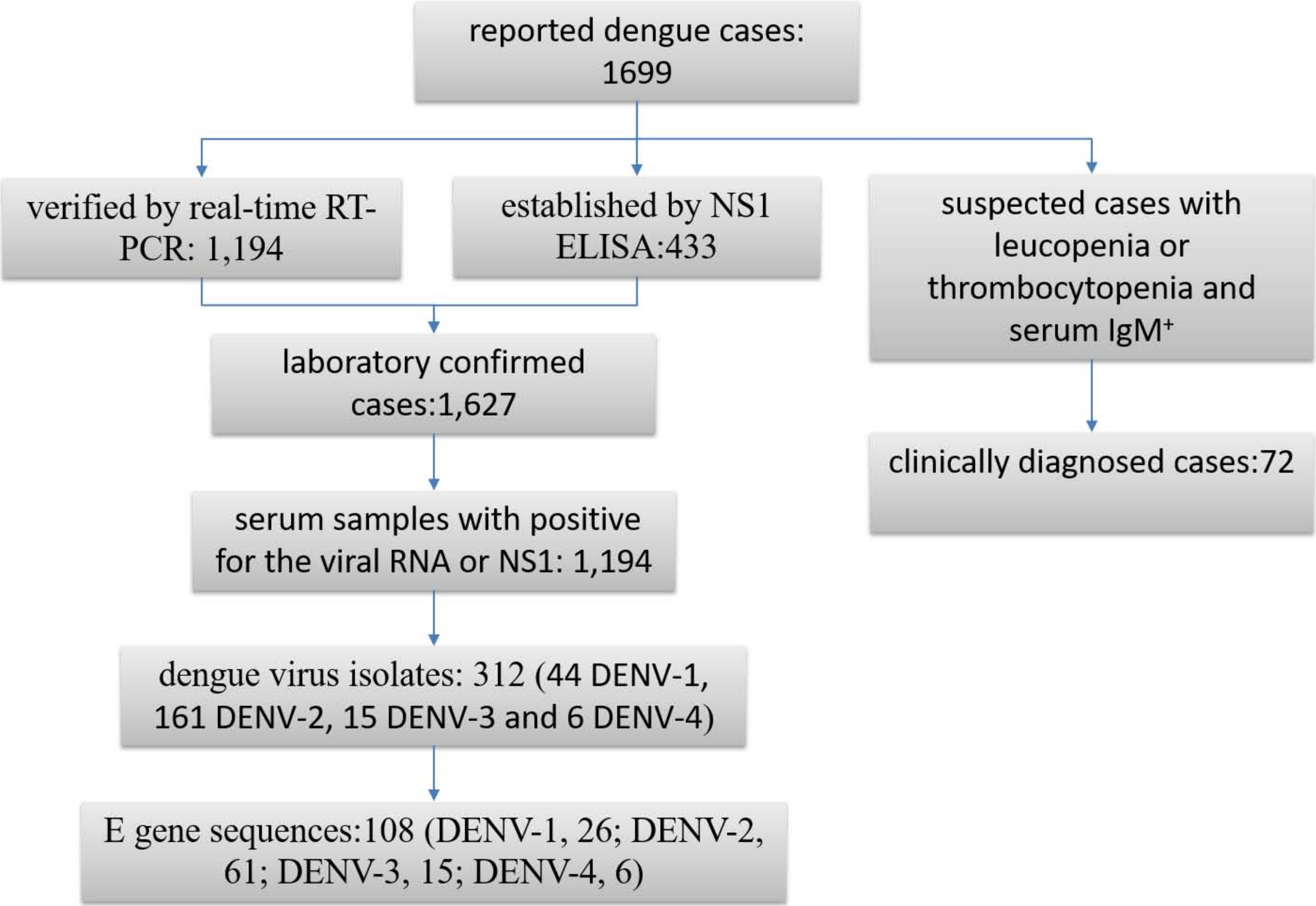
reported dengue cases: 1699
verified by real-time RT-PCR: 1,194
established by NS1 ELISA:433
suspected cases with leucopenia or thrombocytopenia and serum IgM+
laboratory confirmed cases:1,627
clinically diagnosed cases:72
serum samples with positive for the viral RNA or NS1: 1,194
dengue virus isolates: 312 (44 DENV-1, 161 DENV-2, 15 DENV-3 and 6 DENV-4)
E gene sequences:108 (DENV-1, 26; DENV-2, 61; DENV-3, 15; DENV-4, 6)
